# Extracellular Vesicles in Reprogramming of the Ewing Sarcoma Tumor Microenvironment

**DOI:** 10.3389/fcell.2021.726205

**Published:** 2021-09-17

**Authors:** Manideep C. Pachva, Horton Lai, Andy Jia, Melanie Rouleau, Poul H. Sorensen

**Affiliations:** ^1^Department of Molecular Oncology, British Columbia Cancer Research Centre, Vancouver, BC, Canada; ^2^Department of Pathology and Laboratory Medicine, University of British Columbia, Vancouver, BC, Canada; ^3^Faculty of Science, University of British Columbia, Vancouver, BC, Canada

**Keywords:** Ewing sarcoma, tumor microenvironment, reprogramming, immunosuppression, metastasis, extracellular vesicles

## Abstract

Ewing sarcoma (EwS) is a highly aggressive cancer and the second most common malignant bone tumor of children and young adults. Although patients with localized disease have a survival rate of approximately 75%, the prognosis for patients with metastatic disease remains dismal (<30%) and has not improved in decades. Standard-of-care treatments include local therapies such as surgery and radiotherapy, in addition to poly-agent adjuvant chemotherapy, and are often associated with long-term disability and reduced quality of life. Novel targeted therapeutic strategies that are more efficacious and less toxic are therefore desperately needed, particularly for metastatic disease, given that the presence of metastasis remains the most powerful predictor of poor outcome in EwS. Intercellular communication within the tumor microenvironment is emerging as a crucial mechanism for cancer cells to establish immunosuppressive and cancer-permissive environments, potentially leading to metastasis. Altering this communication within the tumor microenvironment, thereby preventing the transfer of oncogenic signals and molecules, represents a highly promising therapeutic strategy. To achieve this, extracellular vesicles (EVs) offer a candidate mechanism as they are actively released by tumor cells and enriched with proteins and RNAs. EVs are membrane-bound particles released by normal and tumor cells, that play pivotal roles in intercellular communication, including cross-talk between tumor, stromal fibroblast, and immune cells in the local tumor microenvironment and systemic circulation. EwS EVs, including the smaller exosomes and larger microvesicles, have the potential to reprogram a diversity of cells in the tumor microenvironment, by transferring various biomolecules in a cell-specific manner. Insights into the various biomolecules packed in EwS EVs as cargos and the molecular changes they trigger in recipient cells of the tumor microenvironment will shed light on various potential targets for therapeutic intervention in EwS. This review details EwS EVs composition, their potential role in metastasis and in the reprogramming of various cells of the tumor microenvironment, and the potential for clinical intervention.

## Extracellular Vesicles

**FIGURE 1:**
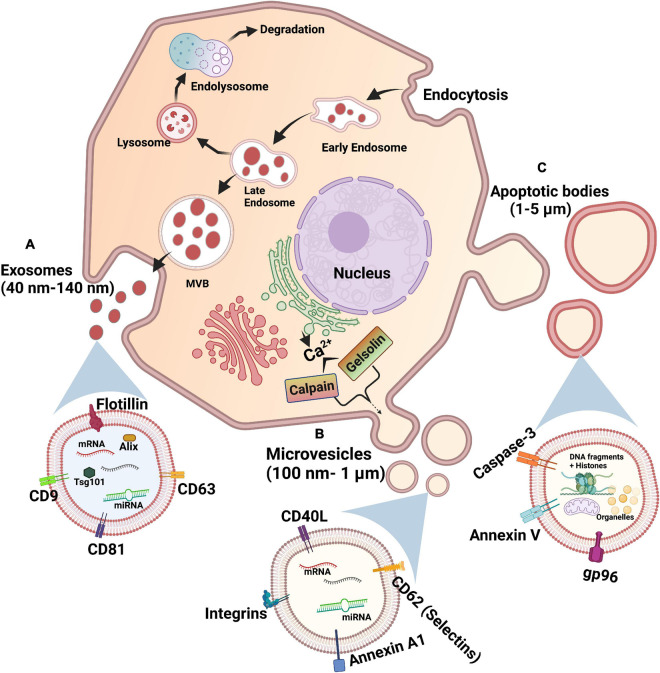
Biogenesis of extracellular vesicles. **(A)** Exosomes: Starts with endocytosis-associated invagination of plasma membrane and forms an early endosome, late endosome, and sorted multivesicular body (MVB) by accumulating intraluminal vesicles, sequentially. MVB fuses with plasma membrane to release exosomes via exocytosis. **(B)** Microvesicles: Calcium ions released into the cytoplasm by endoplasmic reticulum cause the budding of microvesicles from plasma membrane via calpain- or gelsolin-mediated pathways. **(C)** Apoptotic bodies: An apoptotic cell undergoes sequential steps of large protrusions and blebbing of plasma membrane to release apoptotic bodies.

Extracellular vesicles (EVs) are membrane-bound particles in the subcellular size range carrying cargo released from cells. There exists three main types of EVs which are distinguished based on their size, biogenesis, and content: exosomes, microvesicles, and apoptotic bodies (Figure 1; Kerr et al., 1972; Johnstone et al., 1987; Heijnen et al., 1999). Apoptotic bodies are 1–5 μm in size, produced during apoptosis of cells (Table 1 and Figure 1C). Hence, they contain cellular fragments, such as intact organelles and nuclear fractions (Kerr et al., 1972). Unlike the other two types of EVs, apoptotic bodies do not have a known function in intercellular communication under normal physiological conditions (Colombo et al., 2014).

**TABLE 1:** Characteristics of extracellular vesicles subtypes.

Type	Size	Markers	Process of formation
Exosomes	40 nm–140 nm	CD63, CD81, CD82, CD9, Tsg101, and Alix	Endosomal sorting
Microvesicles	100 nm–1 μm	Annexin A1, CD40L, Selectins (CD62), and Integrins	Budding of plasma membrane
Apoptotic bodies	1 μm–5 μm	Annexin V, Caspase-3, and gp96	Protrusions and blebbing of plasma membrane

Exosomes are the smallest of the three types of EVs, with a diameter of 40–140 nm. Exosomes are formed by the budding of the membrane of early endosomes to form intraluminal vesicles (ILVs), turning the endosomes into multivesicular bodies (MVBs) (Table 1 and Figure 1A; Hurley, 2008). Exosomes are released from cells upon the fusion of MVBs with the plasma membrane. The endosomal sorting complexes required for transport (ESCRT) pathway is the key modulator of MVB synthesis in which the ESCRT protein complexes guide intracellular vesicles to their destinations within cells and also direct MVBs toward the plasma membrane (Colombo et al., 2013). Other mechanisms, such as the tetraspanin and ceramide pathways, are known to regulate the formation of ILVs. The tetraspanin CD63 was shown to regulate the sorting of proteins into ILVs and thus the budding of exosomes (van Niel et al., 2011). On the other hand, the sphingolipid ceramide triggers the budding of ILVs (Trajkovic et al., 2008). It was also recently found that caveolin-1, a membrane-associated scaffolding protein, regulates levels of cholesterol in MVBs and hence the biogenesis of exosomes (Albacete-Albacete et al., 2020). Given that ILVs are composed of membrane components, derived from the budding of endosomes via multiple pathways, these proteins and lipids may be present on the surface of exosomes and differ depending on their mechanism of formation. Although the tetraspanins CD63, CD81, and CD9 are not required for exosome biogenesis via the ESCRT-dependent pathway, they are enriched on the surface of exosomes (Escola et al., 1998; Heijnen et al., 1999; Kowal et al., 2016). Hence, these three tetraspanins have been traditionally used as markers for exosome identification (Table 1; Kowal et al., 2016). However, the tetraspanin-enriched exosomes lack cytosolic proteins such as pyruvate kinase and enolase previously thought to be common in exosomes, suggesting the presence of a class of non-classical exosomes devoid of these three tetraspanin markers (Jeppesen et al., 2019). Meanwhile, other previously identified exosomal markers, such as MHC molecules and heat shock protein 70, were refuted since they are also present in other EVs (Kowal et al., 2016).

Microvesicles are a larger class of EVs with a diameter ranging from 100 nm to 1 μm (Heijnen et al., 1999). The synthesis of microvesicles results from the outward budding and shedding of the plasma membrane (Table 1 and Figure 1B; Heijnen et al., 1999). Intracellular Ca^2+^ levels modulate the biogenesis of microvesicles by affecting the plasma membrane and cytoskeleton. Increased concentrations of cytosolic Ca^2+^ activate the protease calpain, leading to the proteolysis of cytoskeletal proteins (Pasquet et al., 1996). Following calpain proteolysis, Ca^2+^ also activates gelsolin, which cleaves actin filaments, enabling cytoskeleton remodeling for the release of microvesicles in lipid rafts or caveolae in the plasma membrane (Sun et al., 1999; del Conde et al., 2005). The proteins on the surface of microvesicles are acquired from the plasma membrane of donor cells, and hence may serve as markers for this type of EVs, including the previously described tetraspanins (Kowal et al., 2016). These proteins may also provide indications of the state of donor cells, as microvesicles from activated and apoptotic cells exhibit different surface markers (Jimenez et al., 2003). Recently, annexin A1 has emerged as a specific protein marker for microvesicles (Table 1). Annexin A1 was found in EVs similar in size to microvesicles that are produced by direct shedding from the plasma membrane of donor cells, but was undetectable in the CD63, CD81, and CD9 tetraspanins-positive exosomes (Jeppesen et al., 2019). Annexin A2 was also discovered in non-exosomal EVs, in a population potentially overlapping with annexin A1 EVs and microvesicles (Jeppesen et al., 2019). Given that EVs are lipid-bound vesicles generated via specific molecular pathways, their lipid composition may serve as a marker for each class of EV (Llorente et al., 2013; Singhto et al., 2019). Indeed, exosomes and microvesicles contain different sets of sphingolipids, exemplified by the validation of ceramide phosphates as a specific marker of urinary exosomes given its undetectable level in urinary microvesicles (Singhto et al., 2019). These novel markers may potentially be used to differentiate the two types of EVs, which is crucial for the characterization of their specific biological functions.

In addition to membrane-associated molecules derived from their membrane of origin, both exosomes and microvesicles contain soluble proteins and nucleic acids as cargos (Valadi et al., 2007; Guescini et al., 2009). Though the specific mechanisms of cargo sorting into EVs are unclear, ESCRT-dependent and ESCRT-independent pathways are believed to contribute to this process (van Niel et al., 2018). Selectivity for specific protein cargo is further regulated by diverse post-translational modifications, such as ubiquitination, sumoylation, and phosphorylation (Blot et al., 2004; Saman et al., 2012; Villarroya-Beltri et al., 2013). Caveolin-1 also controls sorting of exosomal protein cargo, in addition to exosome biogenesis (Albacete-Albacete et al., 2020). Regulation of the RNA content in EVs was shown to be achieved by RNA-binding proteins targeting RNAs to the site of EV synthesis to protect them from degradation or by recognizing specific sequence motifs to control exosomal sorting (Villarroya-Beltri et al., 2013). Meanwhile, the cargo sorting process may be altered in cancer to allow packaging of cancer-specific cargo in EVs (Welton et al., 2010; Ji et al., 2013; Thakur et al., 2014). Aberrant expression of oncogenic molecules like EWS-FLI1 and EV biogenesis molecules like caveolin-1 may deregulate various mechanisms in cells, thereby altering the repertoire of proteins, RNAs, and other biomolecules packaged in their EVs. Additionally, some pathways may potentially be manipulated during tumorigenesis to allow the predominant secretion of one type of EV, such as in hepatocellular carcinoma where long non-coding RNAs are upregulated to enhance exosome secretion (Cao et al., 2019; Yang et al., 2019). Once released by donor cell, the uptake of EVs by a recipient cell may occur through two processes: endocytosis or direct fusion with the plasma membrane of a recipient cell (Morelli et al., 2004; Parolini et al., 2009). Internalization of EVs releases the cargo of donor cells into recipient cells, enabling intercellular communication. In recipient cells, EVs may induce changes in cell phenotype by the transfer of morphogens, such as Hedgehog proteins in humans and Wingless proteins in *Drosophila*, to establish a gradient in tissue, whereby signals state cell maintenance or differentiation (Greco et al., 2001; Martínez et al., 2006). EVs may also transfer receptors such as B1-receptor to allow stimulation of an inflammatory response by agonists (Kahn et al., 2017). In tumorigenesis, EVs may transfer oncogenic proteins as cargo, as depicted by the transmission of EGFRvIII receptor from glioma cells to induce oncogenic activity in other cells (Al-Nedawi et al., 2008). The intercellular communication functions of EVs are also exploited by cancer cells to modify the tumor microenvironment (TME) and normal cells within it.

## Ewing Sarcoma

Ewing sarcoma (EwS) is an aggressive bone and soft tissue sarcoma that is the second most common bone tumor in children and young adults (Grünewald et al., 2018). The peak incidence of EwS occurs at age 15, being more frequent in males than females with a ratio of 3:2. EwS patients with localized disease have a 5-year overall survival rate of ∼65–75% (Gaspar et al., 2015). However, approximately 20–25% of patients present with metastasis at diagnosis, which is associated with a dismal survival rate of <30% which has not improved for decades (Gaspar et al., 2015).

Ewing sarcoma is characterized by the presence of chromosomal translocations, most commonly the *t*(11;22)(q24;q12) chromosomal abnormality found in 85% of EwS cells (Delattre et al., 1992). This results in the fusion of the *FET* family gene *EWSR1* with the *ETS* family gene, *FLI1*. The expression of EWS-FLI1 oncogenic chimeric fusion protein functions as a chimeric transcriptional activator modulating the expression of downstream target genes leading to key tumorigenic events in EwS (Delattre et al., 1992; Ohno et al., 1993). Epigenome profiling shows that EWS-FLI1 functions as part of a crucial chromatin remodeling complex that drives epigenetic reprogramming by inducing and suppressing cell type-specific enhancers to regulate the activation of spatial and temporal components of gene expression in tumors and likely indirectly, in the TME (Tomazou et al., 2015; Sheffield et al., 2017). EWS-FLI1 uses divergent chromatin remodeling mechanisms to induce an open chromatin state of GGAA microsatellites, creating EwS-specific enhancers that physically interact with target gene promoters and/or enhancers, inducing oncogene activation and repressing tumor suppression (Riggi et al., 2014; Johnson et al., 2017). Additionally, EWS-FLI1 is known to regulate a number of non-coding RNAs, which in return could modulate gene expression and therefore could potentially act as epigenetic regulators in EwS tumorigenesis (Grünewald et al., 2018). In the remaining 15% of EwS negative for *EWS-FLI1* fusion transcripts, fusion variants between *EWSR1* and other *ETS* genes are found, mainly involving the *ERG* gene (Sorensen et al., 1994). Other EwS fusion transcripts have also been identified, such as *EWS-ETV1*, *EWS-ETV4*, and *EWS-FEV*, however, these fusions transcripts are rarely seen, compared to EWS-FLI1 and EWS-ERG fusions (Grünewald et al., 2018).

Despite extensive research on EwS genetics, the EwS cell of origin is still unknown. This is mainly due to difficulties in identifying an appropriate model to study the disease. Indeed, a permissive cellular model for the expression of EwS oncogenic chimeric proteins, mainly EWS-FLI and EWS-ERG, is mandatory given that their expression in primary cell lines result in cell death/growth arrest, whereas their expression in primitive cell lines results in incomplete differentiation (Kovar, 2005). However, multiple hypotheses do exist, such as the discovery that cell surface antigens in EwS may have a neuroectodermal lineage, suggesting the presence of a neural crest origin for EwS cells (Lipinski et al., 1986). This finding was supported by the high expression in EwS cells of genes found in neural and fetal tissues (Staege et al., 2004). Evidence also suggests a mesenchymal stem cell (MSC) origin for EwS cells as EWS-FLI1 fusion silencing causes the convergence of the EwS transcriptome toward that of MSCs (Tirode et al., 2007). Unfortunately, there are no known precursor lesions, preventing the study of early stage precancerous cells (Toomey et al., 2010). Studies using genetically modified mouse models were also unsuccessful to identify the molecular events of EwS tumorigenesis since genomic distribution of GGAA repeats is not conserved between human and mouse (Torchia et al., 2003; Gangwal et al., 2008; Lin et al., 2008).

Although systemic chemotherapy has improved survival for patients with localized disease (approximately 70%), overall survival for individuals with metastatic disease remains dismal (<30%) and has not appreciably changed for decades. For instance, results show that high dose chemotherapy with busulfan and melphalan to manage bone metastases has a 36% 5-year event-free survival rate (EFS) (Oberlin et al., 2006). Use of combined myeloablative therapy and stem cell transplant for patients with metastatic disease showed a 20% 2-year EFS (Meyers et al., 2001). Novel therapeutic interventions for EwS are needed to increase this historically low survival rate seen in patients with metastatic disease (Lawlor and Sorensen, 2015). Therapies targeting EwS EVs could shift the current EwS treatment paradigm by abrogating intercellular communications within the EwS TME, thereby representing a highly innovative therapeutic strategy in EwS. In addition, a deep examination of EVs and their content could also be used as a tool to assess therapeutic response, making them very attractive for multiple clinical applications.

## Ewing Sarcoma-Derived Extracellular Vesicles

Research into EVs in the context of EwS is fairly recent, as EwS EVs were first discovered in 2013 by the detection of exosome-associated markers, the tetraspanins CD63 and CD81 shed by EwS cell lines *in vitro* (Miller et al., 2013). Analysis of EwS cell line derived exosomal RNAs revealed a wide range of RNA species, with small RNAs accounting for a large proportion, resulting in an RNA profile distinct from that of donor cells (Miller et al., 2013). Miller et al. (2013) identified the *EWS-FLI1* transcript in EwS exosomes, in addition to 11 transcripts overexpressed in EwS tissue compared to normal tissue, such as transcripts for genes involved in signaling pathways and pluripotency maintenance (Kawano and Kypta, 2003; Khalfallah et al., 2009; Richter et al., 2013). Similarly, *EWS-FLI1* mRNA was detected in microvesicles along with the CD63 marker secreted from three EwS cell lines *in vitro* as well as secreted into the blood of mice harboring EwS xenografts (Tsugita et al., 2013). Evidence was also provided that through microvesicles, fusion transcripts could be transferred between different EwS cell lines (Tsugita et al., 2013). Although there is no evidence of direct involvement of EWS-FLI1 in regulating the biogenesis of exosomes, it was shown that expression of caveolin-1, a central regulator of exosome biogenesis and sorting of exosomal cargo, is directly regulated by EWS-FLI1 (Tirado et al., 2006; Albacete-Albacete et al., 2020). Accordingly, EWS-FLI1 may play a vital role in the biogenesis of exosomes and cargo sorting. However, in a study where EWS-FLI1 was expressed in mesenchymal cells, no increase in exosome yield was reported from these cells, compared to controls (Ventura et al., 2016). This could mean that EWS-FLI1 may only be involved in sorting exosomal cargo but not in the regulation of exosome biogenesis. *EWS-FLI1* transcripts were also detected in microvesicles, which suggests that the fusion protein may also assist in cargo sorting of microvesicles (Tsugita et al., 2013). Nonetheless, the role of EWS-FLI1 in EV biogenesis and cargo sorting needs to be thoroughly investigated. Similar biomolecules are found as cargo of both exosomes and microvesicles, such as *EWS-FLI1* transcripts, making it difficult to identify which type of EV is associated to a specific event of TME reprogramming based on cargo content. This could result from the widespread use of non-specific EV purification methods, leading to co-purification of multiple types of EVs, and from the lack of confirmation of EV type by specific markers. This emphasizes the importance of using a purification method specific to the studied EV type in addition to characterizing the purified EVs using specific markers. This will allow a deeper characterization of the roles of each EV type and their associated cargo in TME reprogramming.

Since monolayer cultures of EwS cell lines are not representative of the TME, a bioengineered tumor model was developed to better simulate the TME to study EwS EVs (Villasante et al., 2016). It was found that both the 3-dimensionality and extracellular matrix (ECM) composition of these bioengineered tumors influenced the size of EwS exosomes. Indeed, exosomes derived from tissue-engineered tumor had similar size distribution as those in EwS patients’ plasma and were significantly smaller than those detected in monolayer cultures, confirming the influence of the TME on EVs properties (Villasante et al., 2016). Consistent with previous studies, the mRNA of enhancer of zeste homolog 2 (EZH2) was detected in bioengineered tumor-derived exosomes, at a level similar to the one of plasma samples from EwS patients, but at a higher level than in exosomes secreted from monolayer cell lines (Miller et al., 2013; Villasante et al., 2016). EZH2 is a histone methyltransferase trimethylating histone H3 at Lys 27, and a transcriptional target of EWS-FLI1, which can form the Polycomb repressive complex 2 associated with the transcriptional repression of tumor suppressors such as p14^ARF^ and p16^INK4a^ (Varambally et al., 2002; Viré et al., 2006). Moreover, contents of exosomes from the bioengineered tumors were successfully transferred to human MSCs, osteoblasts, and osteoclasts, leading to an increase, no change, or a decrease in their EZH2 mRNA levels, respectively (Villasante et al., 2016). As osteoblasts and osteoclasts are present in bone microenvironment, their uptake of EwS EVs may potentially occur *in vivo*. Hence, although EwS-derived exosomes were shown to potentially mediate the interactions between EwS cells and the TME, the functional consequences of these interactions were only investigated recently.

Apart from mRNAs and proteins, miRNAs have also been found in EwS EVs (Table 2). It was found that CD99, a surface marker of EwS cancer cells, could be released through exosomes (Llombart-Bosch et al., 2009; Ventura et al., 2016). CD99 is involved in both physiological and pathological functions such as cell adhesion, migration, and differentiation (Schenkel et al., 2002; Cerisano et al., 2004; Ventura et al., 2016). When CD99 was silenced in EwS cell lines, the secreted exosomes contained increased levels of miR-34a, whose transfer repressed the Notch pathway in EwS recipient cells, inhibiting NF-κB transcriptional activity, and causing their neural differentiation similar to direct CD99 silencing (Ventura et al., 2016). A following study found that CD99-positive exosomes derived from CD99-expressing EwS donor cells led to increased proliferation, increased migration, and poor neural differentiation in EwS recipient cells compared to uptake of exosomes from CD99-silenced EwS donor cells (De Feo et al., 2019). CD99-silenced EwS cells exhibited reduced migratory ability, and their CD99-negative exosomes contained miR-199a-3p, thereby inhibiting growth and migration of recipient cells as seen in other cancers (Fornari et al., 2010; Henry et al., 2010; De Feo et al., 2019).

**TABLE 2:** Markers of extracellular vesicles derived from Ewing sarcoma cells.

Biomarker type	EwS EV Markers	References
RNA (coding)	*NROB1, NKX2.2, STEAP1, LIPI*, and *EWS-FLI1*	Miller et al., 2013; Tsugita et al., 2013
RNA (non-coding)	miR-34a and miR-199a-3p	Ventura et al., 2016; De Feo et al., 2019
Protein	CD99, HINT1, and NGFR	Llombart-Bosch et al., 2009; Ventura et al., 2016; Samuel et al., 2020

In addition to influencing the size of EwS exosomes, it was also found that conditions of the TME impacts the cargo of exosomes, such as miR-210 (Kling et al., 2020). Under hypoxic conditions, proliferation of EwS cells was decreased, resulting in a decrease in the levels of exosomes secretion (Kling et al., 2020). Incubating these exosomes derived from hypoxic cells with EwS cells under normoxic conditions increased sphere formation, indicating that the hypoxic exosomes increased stemness of cancer cells (Kling et al., 2020). Similar to normoxic exosomes, hypoxic exosomes also had a wide range of RNA species, specifically small RNAs, but some were differentially regulated (Miller et al., 2013; Kling et al., 2020). miR-210 was one of the most upregulated miRNAs under hypoxic conditions in EwS cells and exosomes (Fasanaro et al., 2008; Kling et al., 2020). The packaging of miR-210 under hypoxic conditions was shown to be regulated by hypoxia inducible factor-1α, and the effects of miR-210 was proportional to their exosomal levels (Won Kim et al., 2009; Kling et al., 2020). In recipient cells, the acquired exosomal miR-210 induced sphere formation through the silencing of CASP8AP2, a proapoptotic factor whose inhibition promotes recipient EwS cell survival (Won Kim et al., 2009; Kling et al., 2020).

As depicted in multiple cancers, the TME is composed of a diversity of normal cells which are known to influence cancer progression (de Vos van Steenwijk et al., 2013; Kervarrec et al., 2018; Monteran and Erez, 2019). Therefore, EwS EVs are also expected to be absorbed by normal cells, hence having the potential to reprogram them through cargo transfer as seen with EwS recipient cells of miR-210. However, the exact effects of EwS EV uptake on the behaviors of normal cells, as well as the mechanisms underlying their modification, have yet to be directly investigated. Moreover, how EwS-specific cargos are selectively packed into different types of EVs remains to be determined. Given the distinct biogenesis mechanisms of exosomes and microvesicles, in addition to specific sequence motifs as a potential mechanism for sorting RNA cargo, it is expected that EwS-derived exosomes would carry to some extent a different set of cargo than EwS-derived microvesicles, suggesting potential variance in their cell reprogramming ability (Villarroya-Beltri et al., 2013; van Niel et al., 2018). Hence, the proportion of each type of EwS EV and their specific cargos should be considered when evaluating the repertoire of molecules that normal cells may receive from EwS EVs.

## Reprogramming of Ewing Sarcoma Tumor Microenvironment

The TME contains cancer cells and non-malignant cells recruited to the tumor, such as cancer-associated fibroblasts (CAFs), immune cells, vascular cells, pericytes, and adipocytes (Whiteside, 2008; Balkwill et al., 2012). Through their interactions with malignant cells, non-malignant cells acquire tolerance toward the presence of cancer cells and can support tumor growth (Whiteside, 2008; Balkwill et al., 2012). Immune cells recruited to the TME are diverse, including T lymphocytes, B lymphocytes, macrophages, NK cells, and dendritic cells (DCs) (Balkwill et al., 2012). The cells are also surrounded by a non-cellular component, the ECM, which is mainly secreted by CAFs and is composed of collagen, laminin, and fibronectin (Henke et al., 2020).

### Cancer-Associated Fibroblasts

**FIGURE 2:**
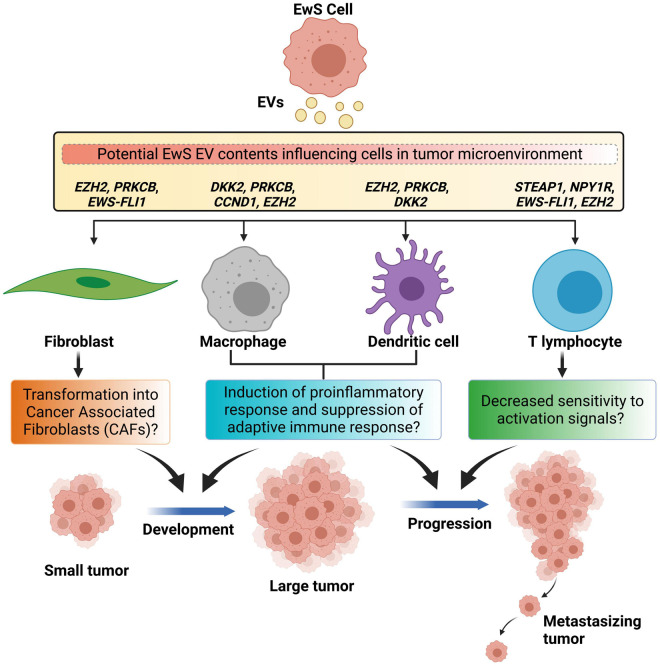
Schematic representation of Ewing sarcoma extracellular vesicle-mediated reprogramming of cells in the tumor microenvironment. Ewing sarcoma (EwS)-derived extracellular vesicles (EVs) may cause transformation of normal fibroblasts into cancer-associated fibroblasts that majorly support the cancer development. EwS-derived EVs may cause tumor-associated macrophages and dendritic cells to produce excessive pro-inflammatory cytokines and cause suppression of tumor specific immune response, and support both the cancer development and its progression. EwS EVs may inhibit T cell-mediated response by decreasing activation associated signals, thereby causing cancer progression.

Cancer-associated fibroblasts within the TME are represented by a heterogeneous population of cells with diverse characteristics, known to facilitate malignant cell migration and invasion by remodeling the ECM (Sugimoto et al., 2006; Sahai et al., 2020). Normal fibroblasts, MSCs, and endothelial cells may give rise to CAFs (Zeisberg et al., 2007; Spaeth et al., 2009; Kojima et al., 2010; Webber et al., 2010). Despite this, the majority of CAFs arise from local cells in the TME, and to a smaller extent from circulating bone marrow-derived cells (Arina et al., 2016). The transformation of normal fibroblasts into CAFs may be assisted by tumor-derived EVs. EVs from osteosarcoma cells were found to convert lung fibroblasts into CAFs *in vitro* (Mazumdar et al., 2020). Similar findings were also reported in breast, bladder, ovarian, and prostate cancers (Webber et al., 2015; Giusti et al., 2018; Goulet et al., 2018; Vu et al., 2019). Hence, EwS EVs could also have the potential to induce the conversion of fibroblasts into CAFs, influencing the levels of CAFs in the TME, though this has yet to be demonstrated (Figure 2).

Ewing sarcoma EVs could also further influence CAF phenotypes and promote cancer progression. One of the components of EwS EVs, *EZH2* transcripts, could potentially alter CAFs, after EV uptake (Miller et al., 2013). In systemic sclerosis (scleroderma), increased levels of EZH2 was detected in dermal fibroblasts, leading to an increased migratory ability (Tsou et al., 2019). Meanwhile, treatment of dermal fibroblasts with DZNep, an EZH2 inhibitor, decreased the expression of genes involved in fibrosis and altered the DNA methylation states of 37 CpG sites in 24 genes (Tsou et al., 2019). Similarly, transfer of *EZH2* transcripts from EwS EVs could increase the level of expression of EZH2 in CAFs, enhancing their ability to migrate. Hence, EwS EVs may potentially convert fibroblasts into CAFs, supporting growth of cancer cells by secreting growth factors and cytokines such as IL-6 known to increase EwS resistance to apoptosis in a paracrine manner (Xing et al., 2010; Lissat et al., 2015). EwS EVs could allow CAFs to migrate away from EwS cells, converting surrounding areas into a more growth-permissive environment with growth factors and cytokines, priming the conditions for EwS cells to expand locally.

Another component of EwS EVs that may influence CAFs functions is represented by protein kinase C beta (*PRKCB*) transcripts (Miller et al., 2013). PRKCB is a subunit of the classical protein kinase C (Wallace et al., 2014), involved in the formation of invadopodia in CAFs but not in normal fibroblasts (Goicoechea et al., 2014). *In vitro*, the invadopodia of CAFs were capable of degrading the ECM, hence assisting CAFs in their remodeling of the TME (Goicoechea et al., 2014). The significance of *PRKCB* transcripts in CAFs on tumor growth is supported by a study from Wallace et al. (2014), who reported that PRKCB expression is increased in CAFs of breast cancer as well as tumors derived from mouse mammary tumor virus in a mouse model (Wallace et al., 2014). Furthermore, knockout of PRKCB in the mouse model reduced the volume and the collagen amounts in the tumor (Wallace et al., 2014). In the case of EwS, transfer of *PRKCB* transcripts into CAFs could also promote the formation of invadopodia, leading to ECM degradation and remodeling. Hence, a modified ECM may facilitate the migration of EwS cells in the TME.

As mentioned, EwS EVs also contain the *EWS-FLI1* fusion mRNA, which may potentially lead to growth arrest in CAFs when translated (Lessnick et al., 2002). When EWS-FLI1 was expressed in primary human fibroblasts, growth arrest was initiated by p53 induction, involving Wnt, receptor tyrosine kinase, and p16-Rb pathways and causing downregulation of genes involved in cell cycle progression (Deneen and Denny, 2001; Lessnick et al., 2002). For the speculated EwS EV-mediated conversion of fibroblasts into CAFs to be successful, p53-mediated growth arrest, triggered by the *EWS-FLI1* in EwS EVs, would need to be bypassed, potentially with the assistance of other components of EwS EVs. EwS EV-mediated reprogramming of CAFs could therefore compromise the associated pathways, but more research is required to understand the implications of their reprogramming.

### T Cells

Through infiltration of tumors, T cells such as CD8^+^ cytotoxic T cells, helper T cells, and regulatory T cells, enter the TME, which may be modulated by specific chemokines (Harlin et al., 2009; Peng et al., 2012; Nagarsheth et al., 2016). Tumor infiltration by activated CD8^+^ T cells is associated with good prognosis in multiple cancers given that these immune cells kill cancer cells displaying tumor antigens on their surface MHC class I proteins (Schumacher et al., 2001; Mahmoud et al., 2011). Helper T cells are also considered tumor-suppressive because they assist in the differentiation of CD8^+^ T cells, while regulatory T cells have been shown to dampen immune response and promote tumor growth (Badoual et al., 2006; Gu-Trantien et al., 2013; Shang et al., 2015). Potential interactions between T cells and EwS EVs and the results of T cell reprogramming hence depend on the proportions of each type of T cell infiltrating the TME (Jordanova et al., 2008).

*EZH2* transcripts in EwS EVs could also alter T cells in the TME (Miller et al., 2013). When EZH2 is absent or inhibited in regulatory T cells, their ability to suppress effector T cells was blocked, increasing intratumoral levels of effector T cells, and reducing tumor growth (Goswami et al., 2018; Wang et al., 2018). In addition, inhibition of EZH2 in effector T cells promoted their differentiation and increased their cytotoxicity, resulting in a stronger immune response against cancer cells (Yang X.-P. et al., 2015; Goswami et al., 2018). These results suggest that the EwS EV-mediated transfer of *EZH2* transcripts may increase the levels of EZH2 in regulatory T cells, thereby compromising immune response effectiveness. For effector T cells, increased levels of EZH2 may suppress their differentiation and cytotoxic functions. Overall, EwS EVs may potentially be involved in the immunosuppression of T cells (Figure 2).

Neuropeptide Y receptor Y1 (NPY1R) transcripts detected in EwS EVs could also inhibit the activation of T cells (Miller et al., 2013). A lack of NPY1R resulted in increased sensitivity of T cells toward activation signals, while increased amounts of NPY1R inhibited T cell proliferation (Wheway et al., 2005). In a study of experimental autoimmune encephalomyelitis, in which Th1 helper T cells are activated by peptides in the central nervous system and produce cytokines, NPY1R was found to suppress the disease (Bedoui et al., 2003). Collectively, these results suggest that NPY1R inhibits T cell activation and functions. As *NPY1R* transcripts are transferred in EwS EVs into helper T cells in the TME, an increased surface expression of NPY1R may potentially decrease the sensitivity of T cells toward activation signals (Figure 2). Therefore, the immune response from T cells would be dampened, providing more favorable conditions for tumor growth and EwS cells survival.

Moreover, transcripts encoding STEAP1, a highly expressed surface protein in EwS cells and a potential EWS-FLI1 fusion protein target gene, were detected within EwS EVs (Grunewald et al., 2012a). STEAP1 expression in recipient cells could result from the transfer of EwS EVs-derived *STEAP1* transcripts, or by subsequent EWS-FLI1 induction following transfer of *EWS-FLI1* transcripts in EwS EVs (Grunewald et al., 2012a; Miller et al., 2013). In EwS cells, knockdown of STEAP1 led to the inactivation of STAT1 (Grunewald et al., 2012a), suggesting that conversely, upregulation of STEAP1 by EwS EVs may lead to STAT1 activation in recipient cells. Meanwhile, STAT1 was involved in the inhibition of CD8^+^ T cell proliferation by type I interferons, and hypothesized to be downregulated by T cells *in vivo* to allow their expansion (Gil et al., 2006; Quigley et al., 2008). If EwS EVs cause STEAP1 expression to activate STAT1 in recipient T cells, EVs may thus reverse the STAT1 downregulation exerted by T cells, hence reinforcing the antiproliferative effects induced by type I interferon potentially present in the TME (Gajewski et al., 2013). While STEAP1 expression in EwS cells is associated with poor outcome (Grunewald et al., 2012b), no association was found between the detection of STEAP1 in prostate cancer EVs and its clinical outcome (Khanna et al., 2021). Hence, the exact role of *STEAP1* transcripts in EwS EVs requires further investigation.

### Tumor-Associated Macrophages

Tumor-associated macrophages (TAMs) are inflammatory components of the stroma in many malignant tumors (Mantovani et al., 2002). TAMs are a major contributor supporting malignant progression by favoring tumor growth, angiogenesis, and metastasis (Ono, 2008). In the TME, TAM population often consists of M2 macrophages that promote tumor progression through activities such as ECM remodeling and adaptive immunity suppression (Sica et al., 2006).

The production of specific cytokines by TAMs could be triggered by EwS EVs and promote tumor growth. Following EwS EV uptake, the potential upregulation of EZH2 in TAMs and its subsequent H3K27me3 activity may facilitate macrophage activation by mediating the interferon gamma-induced repression of anti-inflammatory genes, leading to MyD88-dependent proinflammatory responses (Qiao et al., 2016; Zhang et al., 2018). In *Xenopus* cells, overexpression of dickkopf homolog 2 (DKK2) synergizes with the co-expressed Lrp6 to increase Wnt/β-catenin signaling, initiating a proinflammatory response activation and TAM differentiation into a M2 phenotype (Brott and Sokol, 2002; Bergenfelz et al., 2012; Naskar et al., 2014). Similarly, DKK2 upregulation by EwS EVs (Staege et al., 2004; Miller et al., 2013) may also lead to Wnt5 signaling activation in cells with high levels of Lrp6. Wnt5a induces an immunosuppressive phenotype in macrophages, in which the NF-κB pathway is inhibited and anti-inflammatory cytokine IL-10 is secreted (Bergenfelz et al., 2012). Since IL-10 is associated with immunosuppression (Saraiva and O’Garra, 2010), these findings suggest that the presence of these transcripts in EwS EVs may modulate the repertoire of cytokines produced by TAMs, to reach a fine balance that allows immunosuppression (Figure 2).

Ewing sarcoma EVs could also enhance invasiveness of TAMs to improve their tumor infiltration. The potential upregulation of PRKCB in recipient cells may be caused by the direct transfer of *PRKCB* transcripts within EwS EVs or indirectly by the transfer of *EWS-FLI1* transcripts followed by EWS-FLI1 transcriptional activation of PRKCB (Surdez et al., 2012). Both *in vivo* and *in vitro*, PRKCB was shown to play a role in the maintenance of EwS tumor size and cell survival (Surdez et al., 2012). In 2014, Wallace et al. (2014) found that macrophage infiltration into breast cancer tumor sites is reduced in PRKCB knockout mice, suggesting that PRKCB is crucial for macrophage infiltration which has been associated with poor outcomes in EwS and other cancers (Fujiwara et al., 2011; Yang J. et al., 2015; Yagi et al., 2019). In support of this observation, TAM invasiveness may be modified by the overexpression of another gene transcript found in EwS EVs, such as cyclin D1 (*CCND1*) (Staege et al., 2004; Miller et al., 2013). Cyclin D1 behaves as a regulatory component of cell cycle division and promotes progression through G1-S phase (Fu et al., 2004). Studies showed that bone marrow-derived macrophages (BMMs) from *CCND1*-deficient mice displayed reduced motility, proliferation, and tissue invasiveness, as indicated by an increase in focal complex formation and decreased transmigratory abilities across endothelial cell barriers, resulting in tumor resistance (Neumeister et al., 2003). An enhanced macrophage infiltration into EwS tumors following the transfer of *PRKCB* and *CCND1* transcripts could promote tumor growth (Fujiwara et al., 2011), potentially by increasing the density of macrophages in tumors to amplify the immunosuppressive effects of their proinflammatory cytokines triggered by EZH2 and DKK2 (Figure 2).

### Dendritic Cells

Dendritic cells are a heterogeneous population of antigen presenting cells that arise from CD34^+^ bone marrow stem cells and are known to initiate adaptive immune responses (Banchereau and Steinman, 1998). DCs are also found in the TME where they acquire immunoregulatory functions (Curiel et al., 2003). However, multiple factors such as elevated release of granulocyte macrophage colony stimulating factor by cancer cells in the TME (Serafini et al., 2004) and cancer cell stroma-mediated effects (Zhang et al., 2004) can suppress DC development and tumor regression activity (Fricke and Gabrilovich, 2006).

Through influencing T cell behavior, DCs may also cause immunosuppression in the TME. DKK2 provided in EwS EVs could induce Wnt/β-catenin signaling in CD103^+^ DCs, which is associated with CXCL9/10 depletion in the TME, in turn inhibiting TME T cell infiltration (Brott and Sokol, 2002; Staege et al., 2004; Spranger et al., 2017). Wnt/β-catenin signaling also induces immunological tolerance in DCs within the TME (Hall et al., 2011), possibly through retinoic acid synthesis (Hong et al., 2015). *In vitro* and *in vivo* testing has also shown that increased β-catenin signaling may inhibit the ability of DCs to activate antigen-specific CD8^+^ T cells (Liang et al., 2014). Such factors that lead to decreased adaptive immune responses as a result of DKK2 overexpression in DCs by EwS EVs may account for the immunosuppressive activities found within the EwS TME (Figure 2). Further research is recommended to understand the relation between retinoic acid synthesis and DCs immunological tolerance in the context of EwS.

The uptake of EwS EVs may also influence the differentiation of DCs. PRKCB, derived directly from *PRKCB* transcripts in EwS EVs or from potential EWS-FLI1 activity as a target gene of the fusion protein after EV-mediated transfer of *EWS-FLI1* transcripts, may act as a signaling molecule. This signal could lead to DC differentiation upon their activation by differentiation-inducing stimuli in normal and leukemic progenitor cells (Cejas et al., 2005; Farren et al., 2010; Surdez et al., 2012). Research shows that PRKCB-II transfection into differentiation-resistant DC progenitor cell lines restored their ability to differentiate, suggesting that while DCs in the TME are considered defective, the restoration of differentiation potential through upregulation of PRKCB by EwS EVs might also be possible (Cejas et al., 2005; Balkwill et al., 2012). Furthermore, EZH2 from EwS EVs is potentially oncogenic as it was shown to promote DC neoplasms through EZH2 regulation of adhesion dynamics along with the p-ERK1/2 signaling cascade (Miller et al., 2013; Tian et al., 2018). EZH2 is also involved in DC migration and survival, since its deficiency in mice models impaired adhesion-complex formation and suppressed autoimmune encephalomyelitis, highlighting its complex regulatory role in DCs (Gunawan et al., 2015). As DCs in the TME of EwS may take up the contents of EwS EVs, increased levels of PRKCB and EZH2 expression could potentially initiate abnormal DCs differentiation in the TME and suppress immune response (Figure 2), thus warranting further investigation to elucidate the possible interactions between TME DCs and EwS cells via EwS EVs.

## Extracellular Vesicles in Ewing Sarcoma Metastasis

Through reprogramming various types of cells in the TME, EwS EVs may promote EwS metastasis. Under hypoxic conditions, EwS EVs contain miR-210, which may interact with endothelial cells in the TME (Fasanaro et al., 2008; Zeng et al., 2014). miR-210 has been shown to enable angiogenesis in other cancers, thereby allowing the transport of oxygen and nutrients to enable further growth of the tumor (Yang et al., 2016). Additionally, tumor-derived exosomes in melanoma were found to increase tumor vascular density through transferring MET oncoproteins as cargo into bone marrow-derived cells, converting them into vasculogenic progenitors, and promoting their recruitment into the tumors (Peinado et al., 2012). Newly formed blood vessels also allow dissemination of EVs and cytokines produced by tumor and stromal cells in the TME into the bloodstream, as well as setting the stage for the exit of tumor cells during metastasis (Bielenberg and Zetter, 2015; Guo et al., 2019).

Through access to the bloodstream, EVs are involved in establishing pre-metastatic niche in distant organs (Hood et al., 2011; Costa-Silva et al., 2015; Hoshino et al., 2015). In pancreatic ductal adenocarcinoma, tumor-derived exosomes could be taken up by liver Kupffer cells, ultimately leading to upregulation of fibrosis-associated factors (Costa-Silva et al., 2015). The resulting fibrotic microenvironment, together with the macrophage migration inhibitory factor cargo in the tumor-derived exosomes, then promotes the recruitment of macrophages into the liver for pre-metastatic niche formation in mouse models (Costa-Silva et al., 2015). Meanwhile, EVs from CAFs, which may be a result of tumor-derived EVs mediating conversion of fibroblasts, were able to promote metastasis to the lungs by activating lung fibroblasts in the case of salivary adenoid cystic carcinoma (Kong et al., 2019). Furthermore, compared to tumor-derived EVs, EVs from CAFs presented an even greater ability to remodel the lung ECM for pre-metastatic niche formation (Kong et al., 2019), displaying the significance of reprogramming cells in the TME for metastasis. Fibroblasts may also release EVs to enhance metastatic capacity of tumor cells, as seen in breast cancer in which CD81-positive EVs activate Wnt-planar cell polarity signaling (Luga et al., 2012). It remains to be tested whether the same applies to CAF-derived EVs in EwS and CAFs originated by uptake of EwS EVs.

Ewing sarcoma EVs may also induce the secretion of proinflammatory molecules in distant organs, which facilitates metastasis. In colorectal cancer, when tumor-derived EVs were injected into the bloodstream of mice, the levels of miR-21 in EVs correlated with IL-6 levels in the plasma, which also correlated with liver metastasis (Shao et al., 2018). Furthermore, an association was found between the types of integrins present in EVs and metastasis to specific organs, suggesting that EVs at least partially determine the location of metastasis (Hoshino et al., 2015). Integrins were also found to activate Src in lung fibroblasts by phosphorylation, and led to the expression of S100 proinflammatory molecules by lung fibroblasts and liver Kupffer cells (Hoshino et al., 2015). Considering that integrins are detected in EwS EVs, these results suggest that EwS EVs may determine and prime specific distant organs by triggering inflammation. It is also possible that proinflammatory cytokines released into the plasma by EwS cells directly travel to distant organs (Lissat et al., 2015; Guo et al., 2019).

Moreover, *NPY1R* and *STEAP1* transcripts in tumor-derived EVs may not only act on T cells in the local TME as described above, but also on T cells in distant organs (Miller et al., 2013). As EwS EVs circulate in the blood, activation and proliferation of T cells may also be inhibited, creating an immunosuppressed environment more amenable to pre-metastatic niche formation (Bedoui et al., 2003; Quigley et al., 2008; Grunewald et al., 2012a).

## Clinical Applications of Ewing Sarcoma Extracellular Vesicles

The notion that EVs are key mediators of intercellular communications between EwS cells and other cells in the TME offer several promising clinical avenues, including biomarker identification, disease prognostication, and potential therapeutic strategies. Extracellular vesicles are classified as liquid-based biomarkers, as they are secreted into various biological fluids including blood and urine. From the results of the Grunewald group, of the top five mRNA overexpressed in EwS cells and their EVs that could serve as potential biomarkers (*NR0B1, NKX2.2, STEAP1, LIPI*, and *EWS−FLI1*), *STEAP1* and *EWS-FLI1* mRNAs packed into EwS EV as cargos could serve as biomarkers and as therapeutic targets, by limiting their packaging in EVs or uptake by recipient cells (Miller et al., 2013). Limiting of EV cargo can be accomplished by targeted inhibition of tumorigenic molecules in EwS cells using pharmacological inhibitors, silencing RNAs, and antibodies to surface proteins. Targeting EV biogenesis and their sorting in EwS cells by attenuating the activity of molecules involved in EV biogenesis and sorting components such as caveolin-1 can also limit the packaging of oncogenic cargo in EVs and their associated effects on the TME. STEAP1 is a membrane-bound protein whose major function is to act as an oxidoreductase, involved in mitochondrial transmembrane electron transfer, but may also have a role in exosome biogenesis, like the other members of the STEAP family (Hubert et al., 1999; Sendamarai et al., 2008; Baietti et al., 2012; Miller et al., 2013). If the role of STEAP1 in exosome biogenesis holds true, then targeting STEAP1 in EwS cells may hinder the packaging of oncogenic mRNAs like *EWS-FLI1* and *STEAP1* itself, therefore inhibiting EwS EV-mediated reprogramming of cells in the TME. Therefore, research into this possibility is warranted. Recently, it was shown in prostate cancer that patients’ plasma samples presented significantly higher STEAP1-positive EVs compared to healthy individuals, as analyzed by nanoscale flow cytometry (Khanna et al., 2021). Therefore, presence of STEAP1 in EVs of EwS patients’ samples as well as its association with clinical outcomes should be assessed. Apart from mRNAs, non-coding RNAs including microRNAs packed in EVs may serve as biomarkers. Since there is limited research done in analyzing the microRNA content of EwS EV subpopulations, there may be a scope for finding a panel of microRNAs specifically packed within EwS EVs that could serve as prognostic tools. However, based on research evidence already available, profiling of miR-34a and miR-199a-3p levels in EwS EVs may also serve as a good non-invasive prognostic tool for predicting EwS disease progression, but this needs to be thoroughly examined (Ventura et al., 2016; De Feo et al., 2019). Limiting the packaging of microRNA cargo in Ewing sarcoma EVs by targeting the relevant packaging molecules could also be of great therapeutic value. Although there are several pieces of evidence of the diversity of transcripts present in EwS EVs, the proteomic content of EwS EVs is only starting to be unraveled. Samuel et al. (2020) found that CD99, HINT1, and NGFR can act as biomarkers of small EVs derived from EwS cells. Sorting small EVs derived from EwS cells based on the above protein markers could improve the specificity of EVs derived from heterogeneous cell populations. Previous studies of EwS EVs emphasized the testing of small EVs or exosomes as biomarkers and therapeutic targets; however, another class of EVs, namely microvesicles, could also be of interest in EwS. EwS EVs, with their potential to fuse with recipient cells in the TME, can act as excellent potential carriers of anti-tumor agents for targeted delivery, to limit their TME reprogramming capacity. In addition, EwS EVs carrying similar surface antigens as EwS cells (e.g., CD99, NGFR, and HINT1) can be used for priming of immune cells to increase immunogenicity against EwS surface antigens. Although the usage of EVs as biomarkers and therapeutic targets is a fairly new concept, they present an interesting potential avenue to monitor disease progression and treatment outcomes in EwS patients.

## Concluding Remarks

Although the role of EVs in cancer and other diseases has only been explored very recently, their remarkable ability to act as non-invasive biomarkers, prognostic tools, and therapeutic targets is the reason why there is currently a fast-growing interest in the field. EwS may begin as a localized disease in the bone or soft tissues, but often metastasizes to distant sites such as the lungs, bone marrow, kidneys, and heart. The aggressiveness of EwS can in part be attributed to the capacity of cancer cells to dampen the immune response against them. Therefore, it is of great importance to understand how EwS cells establish an immunosuppressive and cancer-permissive microenvironment, and to identify the mediators of these processes. EVs are vital means for intercellular communications between cancer cells and other cells in the TME, as well as in distant sites, and are potentially key regulators to establish cancer-permissive conditions. Efforts made by Miller et al. (2013) in unraveling the cargo content of EwS EVs found that CD63 and CD81 carrying exosomes from EwS cell lines were enriched with *NR0B1*, *NKX2.2*, *STEAP1*, *LIPI*, and *EWS−FLI1* transcripts. Although these transcripts can be used as markers for EwS-derived EVs, the role of these EwS EV markers in reprogramming of cells in the TME remains to be elucidated. Therefore, it would be useful to profile mRNAs, microRNAs, and proteins and to determine their reprogramming potential, to potentially target EwS EV cargo for therapeutic intervention. In this review, we have focused on reprogramming of cells in the TME of EwS by selected transcripts such as *EZH2*, *PRKCB*, *NPY1R*, and *EWS-FLI1*. However, there could be other transcripts and proteins involved in reprogramming whose presence in EwS EVs and their associated mechanisms are yet to be unraveled. Several studies suggested that EVs of melanoma cells are mediators of metastasis and opened the possibility for potential roles for EVs in other cancers (Hood et al., 2011; Peinado et al., 2012; Costa-Silva et al., 2015; Hoshino et al., 2015). These findings highlight that EV content may vary in different stages of cancer development and correspond to the physiological states of cancer cells (Peinado et al., 2012). Therefore, extensive characterization of EVs from cancer cells at different stages will help develop promising prognostic and diagnostic tools for EwS. Of the pleotropic roles of EVs, their potential to act as biomarkers will change current diagnostic and therapeutic paradigms of EwS. Also, markers like CD99 and NGFR for EwS-derived EVs will help in sorting EwS-specific EVs and their associated cargo and delineating better targets for therapeutic intervention. Moreover, cargo delivery to recipient cells highlights EVs as excellent candidates for carrying various therapeutic molecules, including interfering RNAs, neutralizing antibodies, and possibly even molecular therapeutics. Given the high metastatic rates and aggressiveness of EwS, targeting EVs for therapeutic intervention has the potential to significantly impact survival rates and change the treatment paradigm of this high-risk malignancy.

## Author Contributions

MP conceptualized the manuscript and generated the figures and tables. HL, AJ, and MP carried out literature searches, drafted, and co-wrote the original manuscript. MR and PS were involved in review, revision, editing, and approval of the manuscript before submission. PS was responsible for acquisition of funding for this work. All authors contributed to the article and approved the submitted version.

## Conflict of Interest

The authors declare that the research was conducted in the absence of any commercial or financial relationships that could be construed as a potential conflict of interest.

## Publisher’s Note

All claims expressed in this article are solely those of the authors and do not necessarily represent those of their affiliated organizations, or those of the publisher, the editors and the reviewers. Any product that may be evaluated in this article, or claim that may be made by its manufacturer, is not guaranteed or endorsed by the publisher.
